# Mr. Alder, on the Disordered States of the Lungs

**Published:** 1804-09-01

**Authors:** Thomas Alder

**Affiliations:** Lincoln


### *To the Editors of the Medical and Physical Journal.*

Gentlemen,

THOUGH theoretic productions have in medicine been censured, and are now pretty generally become the objects of contempt, this has not happened from the culpableness or inutility of theory, but from the manner in which such productions were formed. For instead of theorizers going on regularly and with great care, to make inductions from matter of fact, they have spun their doctrines out of their own imaginations. I am now to call your attention to theories, or rather a system, which I conceive formed from facts alone. I began about eight years ago to investigate according to the principles laid down by Lord Verulam; and have finished a system he endeavoured to build, entirely according to the same. Whether it be true must be decided by the judgement of others; but the plan on which which I attempted to proceed cannot I trust but meet with general approbation; and must, *when ably pursued*, lead to great improvements.

The work I allude to is entitled "Outlines of a Treatise on the disordered States of the Lungs." This, in the first place, shews what agents disorder the lungs, in what manner they act, and what disordered states they occasion; it then enquires into the consequences which must arise from those disordered conditions of the pulmonary organs; it finds, particularly, that when the lungs are over-charged with blood, the venæ cavæ cannot properly discharge *their contents;* and consequently, that the venæ jugulares, and venæ hepaticæ, cannot sufficiently discharge *theirs;* and from *these* being overloaded, it discovers various affections (especially named) which must attack the *head* or *liver.* The diseases which arise from a turgescence of the superior cava are made to form one class; and those which spring from a turgescence of the inferior, are formed into another. But a third, and very important class, is formed of those diseases which arise more immediately from an accumulation *in the lungs themselves.* The third part of the work contains such exceptions as may be made against any part of the preceding work, with the reasons why they do not in reality militate against its truth; and such indication of prophylaxis, or treatment of the diseases, as arise from the before-mentioned view of their origin and nature. Notwithstanding the plan I prescribed to myself in forming the above work was such as, if rigidly adhered to, and ably pursued, must have excluded errors; and notwithstanding also the execution took up many years, I am fully sensible error is not impossible; but the work has had my greatest care, and if any others are disposed to point out errors, I shall be very happy to be informed of them.

I have thought it proper to call your attention to the above work, because it was executed upon a new plan; and if true, must lay the foundation *for a new system of medicine.*

I am, &c.

THOMAS ALDER.

*Lincoln, August* 7, 1804.

ACCOUNT

